# Evaluating the Representativeness of US Centricity Electronic Medical Records With Reports From the Centers for Disease Control and Prevention: Comparative Study on Office Visits and Cardiometabolic Conditions

**DOI:** 10.2196/17174

**Published:** 2020-06-03

**Authors:** Olga Montvida, John Epoh Dibato, Sanjoy Paul

**Affiliations:** 1 Melbourne EpiCentre University of Melbourne Melbourne Australia

**Keywords:** electronic medical records, observational study, epidemiology, population health

## Abstract

**Background:**

Electronic medical record (EMR)–based clinical and epidemiological research has dramatically increased over the last decade, although establishing the generalizability of such big databases for conducting epidemiological studies has been an ongoing challenge. To draw meaningful inferences from such studies, it is essential to fully understand the characteristics of the underlying population and potential biases in EMRs.

**Objective:**

This study aimed to assess the generalizability and representativity of the widely used US Centricity Electronic Medical Record (CEMR), a primary and ambulatory care EMR for population health research, using data from the National Ambulatory Medical Care Surveys (NAMCS) and the National Health and Nutrition Examination Surveys (NHANES).

**Methods:**

The number of office visits reported in the NAMCS, designed to meet the need for objective and reliable information about the provision and the use of ambulatory medical care services, was compared with similar data from the CEMR. The distribution of major cardiometabolic diseases in the NHANES, designed to assess the health and nutritional status of adults and children in the United States, was compared with similar data from the CEMR.

**Results:**

Gender and ethnicity distributions were similar between the NAMCS and the CEMR. Younger patients (aged <15 years) were underrepresented in the CEMR compared with the NAMCS. The number of office visits per 100 persons per year was similar: 277.9 (95% CI 259.3-296.5) in the NAMCS and 284.6 (95% CI 284.4-284.7) in the CEMR. However, the number of visits for males was significantly higher in the CEMR (CEMR: 270.8 and NAMCS: 239.0). West and South regions were underrepresented and overrepresented, respectively, in the CEMR. The overall prevalence of diabetes along with age and gender distribution was similar in the CEMR and the NHANES: overall prevalence, 10.1% and 9.7%; male, 11.5% and 10.8%; female, 9.1% and 8.8%; age 20 to 40 years, 2.5% and 1.8%; and age 40 to 60 years, 9.4% and 11.1%, respectively. The prevalence of obesity was similar: 42.1% and 39.6%, with similar age and female distribution (41.5% and 41.1%) but different male distribution (42.7% and 37.9%). The overall prevalence of high cholesterol along with age and female distribution was similar in the CEMR and the NHANES: overall prevalence, 12.4% and 12.4%; and female, 14.8% and 13.2%, respectively. The overall prevalence of hypertension was significantly higher in the CEMR (33.5%) than in the NHANES (95% CI: 27.0%-31.0%).

**Conclusions:**

The distribution of major cardiometabolic diseases in the CEMR is comparable with the national survey results. The CEMR represents the general US population well in terms of office visits and major chronic conditions, whereas the potential subgroup differences in terms of age and gender distribution and prevalence may differ and, therefore, should be carefully taken care of in future studies.

## Introduction

### Background

Large national surveys and registry data provide epidemiological and population-level health information. Although such studies will remain as gold standards in evaluating the health state at a population level, the more recent development of large real-world data (RWD) from electronic medical records (EMRs) and claims data for therapeutic management and population-level safety evaluations provide additional and unique opportunities to expand our understanding in a broad class of clinical, epidemiological, and public health–related questions [1-6].

EMR data are collected during routine medical care, offering the opportunity to investigate clinical questions from a real-world perspective. Although randomized clinical trials (RCTs) allow the evaluation of the safety and efficacy of interventions in a design-led population, the EMR-based studies allow for comparative effectiveness and safety studies, apart from revolutionizing the approach to efficient pharmacovigilance. RWD-based studies also provide opportunities to explore clinical questions in populations that are often excluded from RCTs, such as pregnant, older, or comorbid patients. Furthermore, real-world studies allow us to investigate questions that may be unethical for testing in RCTs. EMRs are also used to track how clinical guidelines are implemented in real-world practices and to research the quality of clinical care.

The epidemiological value of EMR-based research directly depends on the size of the EMR network. Several EMR systems were implemented at the national level, and most familiar representatives include databases from the United States, the United Kingdom, Sweden, Norway, and Denmark [7-10]. The representativeness of some of these databases in terms of demographics and chronic and rare diseases has been shown in some studies [8,9,11-14].

Apart from health research based on data from individual practices, pharmacies, insurers, claims, or prescriptions, the MarketScan Commercial Claims and Encounters Database, owned by Truven Health Analytics, is one of the most commonly used data source for health research in the United States [15,16]. The Veteran Affairs–integrated health care system is another widely used data source in the United States [17,18]. One of the oldest primary and ambulatory EMR systems in the United States is the Centricity Electronic Medical Record (CEMR), owned by General Electric, which provides an opportunity for research using deidentified data on more than 45 million patients from all states of the United States [19,20].

The CEMR database has been extensively used for health outcome academic research worldwide in the fields of diabetes [21-26], cardiovascular research [27-31], obesity [32-34], inflammatory diseases [35-38], mental health [39-41], and other diseases [42,43]. To draw meaningful inferences from such studies and to generalize the results, it is essential to understand the underlying population. For instance, using the CEMR, Montvida et al [44] described trends in the antidiabetic drug prescription patterns during the years 2005-2016. However, the study should be interpreted in the light of overall representativity of the CEMR with respect to diabetes as well as gender and ethnic differences, as all these factors affect drug choices.

To the best of our knowledge, only two studies have investigated the representativeness of the CEMR database [14,45]. Brixner et al [14] evaluated the BMI and laboratory data from the CEMR in 2003 to 2004 in comparison with the US national health surveys. Crawford et al [45] compared the National Ambulatory Medical Care Survey (NAMCS) with CEMR’s office visits during 2005 and concluded that CEMR data provide a more accurate estimate of the distribution of diagnoses in ambulatory visits in the United States.

### Aims

Given the significant increase in CEMR coverage since the last report was published and exponentially increasing volume of RWD-based research, we aimed to repeat and expand the exploration of the generalizability and representativity of the CEMR database with two of the most widely used and relevant survey results from the United States. Specifically, the goals of this study were to compare (1) patient demographics in the CEMR with the NAMCS and (2) the prevalence of obesity, hypertension, high total cholesterol, and diabetes in the CEMR with the respective reports based on the National Health and Nutrition Examination Surveys (NHANES).

### Data

#### Centricity Electronic Medical Record

The CEMR incorporates patient-level data from independent physician practices, academic medical centers, hospitals, and large integrated delivery networks in the United States. The Medical Quality Improvement Consortium is a rapidly growing community that contributes deidentified clinical data to the CEMR research database to enable quality improvement, benchmarking, and population-based medical research [40,46]. With an average follow-up of 4.5 years, the CEMR research database covers more than 35,000 health care providers from all states of the United States, where approximately 70% are primary care providers. Longitudinal EMRs were available for more than 45 million individuals from 1995 to September 2018, with comprehensive patient-level information on demographics, anthropometric measures, disease events, medications, and clinical and laboratory measures. The database has been extensively used in academic research [14,44,45].

#### The National Ambulatory Medical Care Survey

A report based on the 2016 NAMCS data was used in this study [47]. The excerpts from the Centers for Disease Control and Prevention (CDC) website are presented in the following two paragraphs [47,48].

The NAMCS is a national survey designed to meet the need for objective, reliable information about the provision and the use of ambulatory medical care services in the United States. The findings were based on a sample of visits to nonfederally employed office-based physicians who are primarily engaged in direct patient care. Physicians specializing in anesthesiology, pathology, and radiology were excluded from the survey. Each physician was randomly assigned to a 1-week reporting period. During this period, data for a systematic random sample of visits were recorded by Census interviewers using an automated patient record form (PRF) developed for that purpose.

The 2016 NAMCS sampling design used a stratified two-stage sample, with physicians selected in the first stage and visits in the second stage. The 2016 NAMCS sample included 3699 physicians. Of the 2080 in-scope (eligible) physicians, 677 completed PRFs in the study. Of the 677 physicians who completed PRFs, 536 participated fully or adequately (ie, at least one half of the expected PRFs were submitted, based on the total number of visits during the reporting week) and 141 participated minimally (ie, fewer than half of the expected number of PRFs were submitted). Within physician practices, data were abstracted from medical records for up to 30 sampled visits during a randomly assigned 1-week reporting period. In total, 13,165 PRFs were submitted. The participation rate—the percentage of in-scope physicians for whom at least one PRF was completed—was 39.3%. The response rate—the percentage of in-scope physicians for whom at least one half of their expected number of PRFs was completed—was 32.7%. Among the 4 census regions, response rates ranged from 24.6% to 40.0%.

#### The National Health and Nutrition Examination Survey

The NHANES is a program of studies designed to assess the health and nutritional status of adults and children in the United States [49]. The survey consists of interviews conducted in participants’ homes, standardized physical examinations in mobile examination centers, and laboratory tests on blood and other specimens.

Individual reports produced by the CDC based on the NHANES 2015-2016 data were used to compare the prevalence of obesity [50], hypertension [51], and high total cholesterol [52]. The latest CDC report for diabetes prevalence was based on the NHANES 2013-2016 data [53].

## Methods

Data from the CEMR were matched on methods to individual CDC reports as close as possible.

### Office Visits

Data on percent distribution of office visits and number of office visits per 100 patients by various subgroups from the NAMCS were compared with similar data from the CEMR. All office visits in 2016 for patients with nonmissing age and sex from the CEMR were aggregated to match the NAMCS report as close as possible.

### Obesity

In the NHANES, obesity was defined as a BMI of 30 kg/m^2^ or greater for adults aged 20 years and older [50].

In the CEMR, the proportion of obese people was estimated among people aged older than 20 years and with at least one BMI measure (direct or estimated using weight and height) during the years 2015-2016. Women who had pregnancy-related records before or within the estimated time frame were excluded.

### Hypertension

In the NHANES, systolic blood pressure (SBP) of 140 mm Hg or greater, diastolic blood pressure (DBP) of 90 mm Hg or greater, or currently taking medication to lower high blood pressure were defining hypertension for people aged 18 years and older [51].

In the CEMR, the proportion of patients with hypertension during the years 2015-2016 was estimated among people aged older than 18 years. On average, patients had 4 blood pressure measures during a 2-year time frame. Those who had an average of available measures for SBP of 140 mm Hg or greater, those who had an average of available measures for DBP of 90 mm Hg or greater, or those who were taking medication to lower high blood pressure during the respective time frame were considered to have hypertension. Blood pressure–lowering medications included diuretics, peripheral vasodilators, beta blockers, calcium channel blockers, angiotensin-converting enzyme inhibitors, angiotensin II receptor blockers, and other agents acting on the renin-angiotensin system. Only medications that are indicated to lower blood pressure were preserved within these drug classes.

### High Total Cholesterol

In the NHANES, proportions of participants aged 20 years and older with high total cholesterol (≥240 mg/dL) were reported [52]. In the CEMR, among people aged older than 20 years and with at least one available cholesterol measure, the proportions of those with total cholesterol of 240 mg/dL or greater were estimated during the years 2015-2016.

### Diabetes

In the NHANES, participants were classified as having diagnosed diabetes if they answered “yes” to the question, “Other than during pregnancy, have you ever been told by a doctor or health professional that you have diabetes or sugar diabetes?” [53]. Participants were classified as having undiagnosed diabetes if they did not report a diagnosis of diabetes by a health care provider and their fasting (8-24 hours) plasma glucose level was 126 mg/dL or greater or their hemoglobin A_1c_ (HbA_1c_) level was 6.5% or greater. Participants were randomly assigned to a morning, afternoon, or evening examination. Fasting plasma glucose data from the morning examination (after an 8- to 24-hour fast) were used to define total and undiagnosed diabetes.

In the CEMR, an algorithm to identify patients with diabetes was developed on the basis of (1) diabetes diagnostic codes (International Classification of Diseases and SNOMED), (2) antidiabetic medication prescription patterns, (3) availability of 2 measurements of HbA_1c_ level of 6.5% or greater or fasting blood glucose level of 126 mg/dL or greater or random blood glucose level of 200 mg/dL or greater within 1 year, and (4) keyword searching procedures for diabetic-related terms from the clinical notes of every patient. The algorithm was developed on the basis of clinical guidelines and machine learning suggestions described by Adjah et al [54] for a database from the United Kingdom. Patients who were prescribed metformin for polycystic ovary syndrome were detected and excluded. In the case of nondefinite diabetes subtype, a patient’s age and insulin and noninsulin prescription patterns were used to distinguish subtypes. The off-label use of antidiabetic drugs was not explored. For analyses in this study, patients with prediabetes and gestational diabetes were excluded. The proportion of patients with coded and noncoded diabetes was estimated among adults aged 20 years and older and who were active in the CEMR during the years 2013-2016.

### Statistical Methods

Proportional distributions between the CEMR and the NAMCS and NHANES were compared using the chi-square test, where appropriate. Office visit estimates in the NAMCS report are based on sample data weighted to produce annual national estimates and include SEs. All estimates in the NHANES reports were age adjusted using the 2000 US Census population. For the NAMCS and the NHANES estimates, 95% CIs were calculated using the available SE estimates from the reports.

Crude estimates from the CEMR data were calculated and presented in this study, 95% CI for percentages were calculated based on binomial distribution assumption, and 95% CIs for number of visits per 100 persons per year were calculated assuming a Poisson distribution.

Statistical equivalence for the pairwise comparisons of proportional distributions, where appropriate, was evaluated using the two one-sided test (TOST) of equivalence [55,56] with a ±2.5, ±5, and ±7.5 percentage point equivalence margins. Using population summary statistics on mean, SD, and total number of office visits, the TOST procedure available in SAS 9.4 (SAS Institute Inc) was employed.

## Results

### Office Visits

The NAMCS estimated 883,725,000 office visits in the United States in 2016. In the CEMR, 29,207,860 office visits in 2016 occurred for patients with nonmissing age and sex. In the NAMCS and the CEMR, sex distribution was similar (equivalent at 2.5 percentage point margin), where 58.0% (95% CI 56.2-59.8) and 59.8% (95% CI 59.8-59.8) of all visits were by females (Table 1). The number of visits per 100 females per year was similar in the NAMCS and the CEMR: 315.0 (95% CI 291.5-338.5) versus 294.6 (95% CI 294.5-294.8), whereas the number of visits per 100 males per year was lower in the NAMCS compared with the CEMR: 239.0 (220.6-257.4) versus 270.8 (270.6-270.9).

**Table 1:** Patient characteristics at office visits (National Ambulatory Medical Care Surveys estimated visits, N=883,725,000 and Centricity Electronic Medical Record total visits, N=29,207,860).

Characteristics^a^		National Ambulatory Medical Care Surveys 2016		Centricity Electronic Medical Record 2016	
		Percent distribution (95% CI)	Number of visits per 100 persons per year (95% CI)	Percent distribution (95% CI)	Number of visits per 100 persons per year (95% CI)
All visits		100 (N/A)^b^	277.9 (259.3-296.5)	100 (N/A)	284.6 (284.4-284.7)
**Age (years)**					
	<15	17.7 (14.6-20.8)	257.4 (205.5-309.3)	11.2 (11.2-11.2)^c^	276.5 (276.2-276.8)
	15-24	7.4 (6.6-8.2)	153.0 (132.6-173.4)	7.5 (7.4-7.5)	244.8 (244.5-245.1)
	25-44	19.3 (17.5-21.1)	205.4 (184.4-226.4)	20.0 (20.0-20.0)	272.1 (271.9-272.4)
	45-64	28.5 (26.7-30.3)	301.9 (275.0-328.8)	30.9 (30.9-31.0)	280.3 (280.1-280.4)
	65-74	15.0 (13.8-16.2)	465.2 (419.7-510.7)	16.3 (16.3-16.3)	303.3 (303.1-303.6)
	≥75	12.1 (10.9-13.3)	546.8 (491.3-602.3)	14.1 (14.1-14.1)	329.1 (328.8-329.4)
**Female**					
	Total	58.0 (56.2-59.8)	315.0 (291.5-338.5)	59.8 (59.8-59.8)	294.6 (294.5-294.8)
	<15	8.4 (6.6-10.2)	247.7 (186.9-308.5)	5.4 (5.3-5.4)^d^	274.4 (274.0-274.8)
	15-24	4.6 (3.8-5.4)	194.4 (160.9-227.9)	4.9 (4.9-4.9)	267.3 (266.8-267.7)
	25-44	13.4 (12.0-14.8)	281.8 (249.1-314.5)	13.8 (13.8-13.8)	293.8 (293.5-294.0)
	45-64	16.4 (15.0-17.8)	337.2 (303.1-371.3)	18.2 (18.2-18.2)	286.5 (286.3-286.8)
	65-74	8.0 (7.2-8.8)	467.9 (415.6-520.2)	9.2 (9.2-9.2)	310.0 (309.6-310.4)
	≥75	7.1 (6.3-7.9)	549.9 (484.2-615.6)	8.3 (8.2-8.3)	335.2 (334.8-335.6)
**Male**					
	Total	42.0 (40.2-43.8)	239.0 (220.6-257.4)	40.2 (40.2-40.2)	270.8 (270.6-270.9)
	<15	9.4 (7.8-11.0)	266.7 (216.3-317.1)	5.9 (5.8-5.9)^d^	278.5 (278.1-278.9)
	15-24	2.7 (2.3-3.1)	112.3 (93.9-130.7)	2.5 (2.5-2.5)	210.0 (209.5-210.5)
	25-44	5.9 (5.1-6.7)	126.9 (108.7-145.1)	6.2 (6.2-6.2)	233.6 (233.3-233.9)
	45-64	12.1 (10.9-13.3)	264.4 (234.4-294.4)	12.7 (12.7-12.7)	271.7 (271.5-272.0)
	65-74	6.9 (6.1-7.7)	462.2 (410.1-514.3)	7.1 (7.1-7.1)	295.1 (294.7-295.5)
	≥75	5 (4.4-5.6)	542.4 (479.5-605.3)	5.9 (5.9-5.9)	320.8 (320.4-321.3)
**Ethnicity**					
	White	83.8 (82.0-85.6)	302.3 (281.1-323.5)	85.7 (85.7-85.7)	289.7 (289.6-289.8)
	Black or African American	10.6 (9.2-12.0)	224.3 (192.2-256.4)	10.7 (10.7-10.7)	293.7 (293.3-294.0)
	Other race	5.6 (4.4-6.8)	158.1 (123.8-192.4)	3.6 (3.6-3.6)	272.7 (272.2-273.3)
**Geographic region**					
	Northeast	20.8 (18.1-23.5)	332.0 (285.4-378.6)	21.7 (21.7-21.7)	292.0 (291.8-292.3)
	Midwest	21.2 (18.5-23.9)	279.6 (242.8-316.4)	16.3 (16.3-16.4)^d^	286.4 (286.1-286.6)
	South	36 (32.5-39.5)	264.7 (229.8-299.6)	43.1 (43.1-43.2)^c^	281.7 (281.5-281.8)
	West	22 (19.3-24.7)	257.6 (221.7-293.5)	18.8 (18.8-18.8)^d^	283.4 (283.2-283.7)

^a^Pairwise comparisons for the equivalence of percent distributions between National Ambulatory Medical Care Surveys and Centricity Electronic Medical Record were conducted using the two one-sided test. The unmarked categories were all equivalent at a 2.5 percentage point margin.

^b^N/A: not applicable.

^c^Not equivalent at both 2.5 and 5.0 percentage point margins.

^d^Not equivalent at 2.5 percentage point margin.

Looking into office visits’ percent distribution by age, it was similar between data sources (*P*=.22 for overall and *P*=.23 by age and sex). The CEMR contains fewer visits by younger patients: the age group <15 years did not reach equivalence at the 5 percentage point equivalence margin for overall comparison and was not equivalent at the 2.5 percentage point margin in comparisons by sex. Age groups of 15-24/25-44 years were equally likely to have a visit with proportions of 7.4% (95% CI 6.6-8.2)/19.3% (95% CI 17.5-21.1) and 7.5% (95% CI 7.4-7.5)/20.0% (95% CI 20.0-20.0) in the NAMCS and the CEMR, respectively. Overall, there were 277.9 (95% CI 259.3-296.5) and 284.6 (95% CI 284.4-284.7) office visits per 100 persons in 2016 in the NAMCS and the CEMR, respectively. Younger patients had similar numbers of visits per year per 100 persons: 257.4 (95% CI 205.5-309.3) and 276.5 (95% CI 276.2-276.8) in the NAMCS and the CEMR, respectively; middle age groups (15-44 years) had significantly fewer visits in the NAMCS; and patients older than 65 years had significantly more visits in the NAMCS compared with the CEMR (*P*<.05).

The overall ethnicity distribution was similar between the NAMCS and CEMR groups (*P*=.20). The proportion of visits by white among all visits were similar in the CEMR (85.7% [95% CI 85.7-85.7]) and NAMCS (83.8% [95% CI 82.0-85.6]; equivalent at 2.5 percentage point margin). The number of visits per 100 persons per year was also similar: CEMR, 289.7 (95% CI 289.6-289.8); and NAMCS, 302.3 (95% CI 281.1-323.5). Although the share of office visits by black or African Americans was similar in both data sources (11%, equivalent at 2.5 percentage point margin), there were significantly fewer office visits per 100 persons per year in NAMCS compared with CEMR in this ethnic group: 224.3 (95% CI 192.2-256.4) versus 293.7 (95% CI 293.3-294.0); *P*<.05.

The geographical distribution of office locations in the CEMR and the NAMCS was similar (Table 1; *P*=.23), with underrepresented Midwest and West (not equivalent at 2.5 percentage point margin) and overrepresented South in the CEMR compared with the NAMCS (not equivalent at 5 percentage point margin).

### Prevalence of Chronic Conditions

#### Obesity

Compared with the NHANES 2015-2016 report, the total obesity prevalence in adults was similar: 39.6% (95% CI 36.5-42.7) in the NHANES and 42.1% (95% CI 42.0-42.1) in the CEMR (Table 2; equivalent at 2.5 percentage point margin). Subgroup analyses revealed a lower proportion of obese males in the NHANES compared with the CEMR: 37.9% (95% CI 33.4-42.4) versus 42.7% (95% CI 42.7-42.8; not equivalent at 2.5 percentage point margin, equivalent at 5 percentage point margin), with the poorest agreement between males aged 40 to 59 years (not equivalent at 7.5 percentage point margin).

#### Hypertension

During the years 2015-2016, hypertension prevalence in adults was higher in the CEMR than in the NHANES: 33.5% (95% CI 33.5-33.5) versus 29.0% (95% CI 27.0-31.0; Table 2; not equivalent at 2.5 percentage point margin and equivalent at 5 percentage point margin). However, in the CEMR, the prevalence was significantly lower for older patients (aged 60+ years), not equivalent at 7.5 percentage point margin.

#### High Total Cholesterol

The proportions of adults with high total cholesterol were similar across the NHANES and the CEMR, with a total of 12.4% (Table 2). Although the 95% CI of the proportion of males with high total cholesterol indicated a significant difference between the NHANES (95% CI 9.6%-13.2%) and the CEMR (95% CI 9.3%-9.3%), the proportions appeared to be equivalent at 2.5 percentage point margin based on the TOST.

#### Diabetes

In the NHANES, the proportion of adults with diabetes during the years 2013-2016 was reported to be 14.0% (95% CI 12.8-15.2); among them, 9.7% (95% CI 8.7%-10.7%) were diagnosed and 4.3% (95% CI 3.5%-5.1%) were undiagnosed (Table 3). In the CEMR, 10.1% of adults were estimated to have diabetes, 8.1% of adults had a diagnostic code, and 2% of adults were without (false negatives). Comparing total diabetes estimates in the CEMR with *diagnosed* in the NHANES, the total and by gender prevalence was similar in both data sources (equivalent at 2.5 percentage point margin), and there were fewer seniors (aged 60+ years) with estimated diabetes in the CEMR compared with the NHANES: 16.4% (95% CI 16.4-16.4) versus 21.0% (95% CI 18.5-23.5; not equivalent at 2.5 percentage point margin and equivalent at 5 percentage point margin).

**Table 2:** Prevalence of chronic conditions in adult populations in the National Health and Nutrition Examination Surveys and the Centricity Electronic Medical Record.

Characteristics^a^		National Health and Nutrition Examination Surveys 2015-2016			Centricity Electronic Medical Record 2015-2016		
		Total, % (95% CI)	Male, % (95% CI)	Female, % (95% CI)	Total, % (95% CI)	Male, % (95% CI)	Female, % (95% CI)
**Obesity**							
	All adults	39.6 (36.5-42.7)	37.9 (33.4-42.4)	41.1 (38.0-44.2)	42.1 (42.0-42.1)	42.7 (42.7-42.8)^b^	41.5 (41.5-41.6)
	Adults aged 20-39 years	35.7 (32.0-39.4)	34.8 (29.3-40.3)	36.5 (33.4-39.6)	34.8 (34.8-34.8)	34.5 (34.5-34.6)	35.0 (35.0-35.0)
	Adults aged 40-59 years	42.8 (37.7-47.9)	40.8 (35.1-46.5)	44.7 (38.6-50.8)	47.9 (47.9-47.9)^c^	49.4 (49.4-49.5)^d^	46.7 (46.7-46.7)
	Adults aged 60+ years	41.0 (37.3-44.7)	38.5 (35.0-42.0)	43.1 (37.6-48.6)	41.2 (41.2-41.2)	41.6 (41.6-41.6)^b^	40.9 (40.9-40.9)
**Hypertension**							
	All adults	29.0 (27.0-31.0)	30.2 (27.5-32.9)	27.7 (25.7-29.7)	33.5 (33.5-33.5)^b^	36.1 (36.0-36.1)^c^	31.6 (31.6-31.7)^b^
	Adults aged 20-39 years	7.5 (5.5-9.5)	9.2 (6.5-11.9)	5.6 (3.4-7.8)	9.6 (9.6-9.6)	11.3 (11.2-11.3)	8.6 (8.6-8.6)^b^
	Adults aged 40-59 years	33.2 (29.9-36.5)	37.2 (31.5-42.9)	29.4 (25.5-33.3)	30.6 (30.6-30.7)^b^	33.4 (33.4-33.5)^b^	28.6 (28.5-28.6)
	Adults aged 60+ years	63.1 (59.0-67.2)	58.5 (54.2-62.8)	66.8 (61.7-71.9)	52.2 (52.2-52.2)^d^	53.4 (53.4-53.4)^d^	51.3 (51.3-51.3)^d^
**High total cholesterol**							
	All adults	12.4 (10.7-14.1)	11.4 (9.6-13.2)	13.2 (11.0-15.4)	12.4 (12.3-12.4)	9.3 (9.3-9.3)	14.8 (14.8-14.8)
	Adults aged 20-39 years	7.9 (6.4-9.4)	9.1 (7.2-11.0)	6.7 (4.2-9.2)	7.4 (7.3-7.4)	9.5 (9.5-9.5)	5.8 (5.8-5.9)
	Adults aged 40-59 years	17.1 (14.1-20.1)	16.5 (12.8-20.2)	17.7 (14.9-20.5)	15.1 (15.1-15.1)	12.9 (12.9-12.9)^b^	17.0 (17.0-17.0)
	Adults aged 60+ years	12.5 (11.1-13.9)	6.9 (4.3-9.5)	17.2 (14.4-20.0)	11.9 (11.9-11.9)	6.1 (6.1-6.1)	16.6 (16.6-16.6)

^a^Pairwise comparisons for the equivalence of percent distributions between National Ambulatory Medical Care Surveys and the Centricity Electronic Medical Record were conducted using the two one-sided test. The unmarked categories were all equivalent at a 2.5 percentage point margin.

^b^Not equivalent at 2.5 percentage point margins.

^c^Not equivalent at 5.0 percentage point margins.

^d^Not equivalent at 7.5 percentage point margins.

**Table 3:** The prevalence of diabetes in adult populations.

Characteristics	National Health and Nutrition Examination Surveys 2013-2016			Centricity Electronic Medical Record 2013-2016			
	Total, % (95% CI)	Diagnosed, % (95% CI)	Undiagnosed, % (95% CI)		Total, % (95% CI)	Coded, % (95% CI)	Noncoded, % (95% CI)
All	14.0 (12.8-15.2)	9.7 (8.7-10.7)	4.3 (3.5-5.1)		10.1 (10.1-10.1)	8.1 (8.1-8.1)	2.0 (2.0-2.0)
Men	15.9 (14.1-17.7)	10.8 (9.1-12.5)	5.1 (3.7-6.5)		11.5 (11.5-11.5)	9.2 (9.2-9.2)	1.8 (1.8-1.8)
Women	12.2 (10.7-13.7)	8.8 (7.5-10.1)	3.4 (2.6-4.2)		9.1 (9.1-9.1)	7.3 (7.3-7.3)	2.3 (2.3-2.3)
Adults aged 20-39 years	3.5 (2.2-4.8)	1.8 (1.1-2.5)	1.7 (0.9-2.5)		2.5 (2.5-2.5)	2.1 (2.1-2.1)	0.4 (0.4-0.4)
Adults aged 40-59 years	16.3 (13.9-18.7)	11.1 (9.1-13.1)	5.2 (3.6-6.8)		9.4 (9.4-9.4)	7.7 (7.7-7.7)	1.7 (1.7-1.7)
Adults aged 60+ years	28.2 (25.3-31.1)	21.0 (18.5-23.5)	7.2 (5.7-8.7)		16.4 (16.4-16.5)	12.9 (12.9-12.9)	3.5 (3.5-3.5)

## Discussion

### Principal Findings

In this study, we compared the CEMR ambulatory and primary care database with federal reports based on the NAMCS and the NHANES. Although the CEMR and the CDC reports may not be directly compared because of the differences in the data collection nature and methodologies applied, in this study, we have observed that the CEMR is a good source of population health research with regard to cardiometabolic conditions. Specifically, we observed that (1) on average, there were 3 office visits per patient per year in both the NAMCS and the CEMR; (2) the distribution of age at office visits in the CEMR is biased toward older population; (3) although the proportional share of all visits by males and females were similar, females/males had more/fewer visits in the NAMCS, compared with the CEMR; (4) the distribution of office visits were similar for ethnic groups; (5) West regions are underrepresented and South region is overrepresented in the CEMR compared with the NAMCS; (6) compared with the CDC reports based on the NHANES data, the prevalence of obesity and high total cholesterol is similar in the CEMR, whereas hypertension prevalence is 5% higher; and (7) the prevalence of diabetes in the CEMR reflects the diagnosed US population well.

A decade ago, Crawford et al [45] compared CEMR’s office visits during the year 2005 with the NAMCS report. Crawford et al [45] reported that the CEMR had higher proportions of visits by younger patients and by females, compared with the NAMCS. Maintaining similar methods, we observed a reversed trend in age and no difference in the distribution of visits by gender. The overall prevalence in adults with obesity, high cholesterol, and diabetes was similar between the CEMR and the NHANES reports, and the proportion of those with hypertension was higher in the CEMR than in the NHANES. A possible explanation for this result is the ability to track longitudinal information in EMRs, which is especially prominent in chronic conditions. This feature of EMRs provides exceptional opportunities in terms of extending and modifying the classical epidemiologic theory [57].

Closed systems in the West (the Kaiser Permanente [58]) may explain the finding of lower rates for office visits in the West region in the CEMR. Although the CDC reports were adjusted and weighted with the US Census data, CEMR’s crude prevalence estimates of chronic conditions reported in this study are thus biased by geographic regions. This issue should be carefully taken care of in future population health research based on the CEMR data.

### Strengths and Limitations

As with any survey, the results in the NAMCS and the NHANES are subject to sampling and nonsampling errors. Nonsampling errors include reporting and processing errors as well as biases because of nonresponse and incomplete response. In the NAMCS, ethnicity data were missing for 25% of visits [47,48], whereas in the CEMR, unknown race accounted for only 9% of visits.

It is important to highlight that the population that seeks medical care is biased from the general population, and healthy individuals will be underrepresented in any EMR database. For this reason, rather than comparing the CEMR with US Census data, we compared the demographics at the time of office visits in the CEMR with the NAMCS, which is carefully weighted and adjusted for the US population. Although a significant subset of CEMR users is participating in the Medical Quality Improvement Consortium, the CEMR research database is biased toward these practices. The participation and response rates in the NAMCS of less than 40% also introduce a selection bias, although the NAMCS estimates were corrected [47,48].

The limitations of this study include the nonavailability of provider specialty and insurance data in the CEMR for deeper comparisons with the NAMCS. Certain specialties might adopt EMRs at a slower rate, and insured individuals might be overrepresented in commercial databases, compared with the general population. Owing to large cohort sizes in the CEMR, reported CIs are very narrow, and we believe they do not reflect meaningful differences. Adopting TOST with 2.5, 5.0, and 7.5 percentage point equivalence margins for data comparison provides another overview of the equivalence of data sources. As mentioned earlier, CEMR and survey methods are not directly comparable, and we have done our best to match the data as closely as possible. The cardiometabolic prevalence estimates should be interpreted carefully, in the light of methodological and regional differences. However, we believe that the results of this study demonstrate the ability of the CEMR to reflect population health quite well.

### Conclusions

To conclude, epidemiological and population health findings based on the CEMR database might reflect trends in the general US population; however, the possible region, age, and gender biases presented in this study should be treated and interpreted carefully.

## Abbreviations

CDC: Centers for Disease Control and Prevention
CEMR: Centricity Electronic Medical Record
DBP: diastolic blood pressure
EMR: electronic medical record
HbA1c: hemoglobin A1c
NAMCS: National Ambulatory Medical Care Surveys
NHANES: National Health and Nutrition Examination Surveys
PRF: patient record form
RCT: randomized clinical trial
RWD: real-world data
SBP: systolic blood pressure
TOST: two one-sided test

## References

[1] Hemingway H, Asselbergs FW, Danesh J, Dobson R, Maniadakis N, Maggioni A, van Thiel GJ, Cronin M, Brobert G, Vardas P, Anker SD, Grobbee DE, Denaxas S, Innovative Medicines Initiative 2nd Programme‚ Big Data for Better Outcomes‚ BigData@Heart Consortium of 20 Academic and Industry Partners including ESC (2018). Big data from electronic health records for early and late translational cardiovascular research: challenges and potential. Eur Heart J.

[2] Birkhead GS (2017). Successes and continued challenges of electronic health records for chronic disease surveillance. Am J Public Health.

[3] Rassen JA, Bartels DB, Schneeweiss S, Patrick AR, Murk W (2019). Measuring prevalence and incidence of chronic conditions in claims and electronic health record databases. Clin Epidemiol.

[4] Birkhead GS, Klompas M, Shah NR (2015). Uses of electronic health records for public health surveillance to advance public health. Annu Rev Public Health.

[5] Robbins T, Keung SN, Sankar S, Randeva H, Arvanitis TN (2018). Diabetes and the direct secondary use of electronic health records: using routinely collected and stored data to drive research and understanding. Digit Health.

[6] Hecht J (2019). The future of electronic health records. Nature.

[7] Sepper R, Ross P, Tiik M (2011). Nationwide health data management system: a novel approach for integrating biomarker measurements with comprehensive health records in large populations studies. J Proteome Res.

[8] Blak B, Thompson M, Dattani H, Bourke A (2011). Generalisability of the health improvement network (THIN) database: demographics, chronic disease prevalence and mortality rates. Inform Prim Care.

[9] Schmidt M, Schmidt SA, Adelborg K, Sundbøll J, Laugesen K, Ehrenstein V, Sørensen HK (2019). The Danish health care system and epidemiological research: from health care contacts to database records. Clin Epidemiol.

[10] Kosiborod M, Cavender MA, Fu AZ, Wilding JP, Khunti K, Holl RW, Norhammar A, Birkeland KI, Jørgensen ME, Thuresson M, Arya N, Bodegård J, Hammar N, Fenici P, CVD-REAL Investigators and Study Group (2017). Lower risk of heart failure and death in patients initiated on sodium-glucose cotransporter-2 inhibitors versus other glucose-lowering drugs: the CVD-real study (comparative effectiveness of cardiovascular outcomes in new users of sodium-glucose cotransporter-2 inhibitors). Circulation.

[11] Lewis JD, Schinnar R, Bilker WB, Wang X, Strom BL (2007). Validation studies of the health improvement network (THIN) database for pharmacoepidemiology research. Pharmacoepidemiol Drug Saf.

[12] Haynes K, Forde KA, Schinnar R, Wong P, Strom BL, Lewis JD (2009). Cancer incidence in the health improvement network. Pharmacoepidemiol Drug Saf.

[13] Shivade C, Raghavan P, Fosler-Lussier E, Embi PJ, Elhadad N, Johnson SB, Lai AM (2014). A review of approaches to identifying patient phenotype cohorts using electronic health records. J Am Med Inform Assoc.

[14] Brixner D, Said Q, Kirkness C, Oberg B, Ben-Joseph R, Oderda G (2007). Assessment of cardiometabolic risk factors in a national primary care electronic health record database. Value Health.

[15] Adamson D, Chang S, Hansen L (2005). Patient Privacy Rights.

[16] Kulaylat A, Schaefer E, Messaris E, Hollenbeak C (2019). Truven health analytics marketscan databases for clinical research in colon and rectal surgery. Clin Colon Rectal Surg.

[17] Gellad WF, Good CB, Lowe JC, Donohue JM (2010). Variation in prescription use and spending for lipid-lowering and diabetes medications in the veterans affairs healthcare system. Am J Manag Care.

[18] Bohnert KM, Ilgen MA, Louzon S, McCarthy JF, Katz IR (2017). Substance use disorders and the risk of suicide mortality among men and women in the US veterans health administration. Addiction.

[19] Asche CV, Kim J, Kulkarni AS, Chakravarti P, Andersson K (2011). Assessment of association of increased heart rates to cardiovascular events among healthy subjects in the United States: analysis of a primary care electronic medical records database. ISRN Cardiol.

[20] Montvida O, Cai X, Paul SK (2019). Cardiovascular risk factor burden in people with incident type 2 diabetes in the US receiving antidiabetic and cardioprotective therapies. Diabetes Care.

[21] Unni S, Wittbrodt E, Ma J, Schauerhamer M, Hurd J, Ruiz-Negrón N, McAdam-Marx C (2018). Comparative effectiveness of once-weekly glucagon-like peptide-1 receptor agonists with regard to 6-month glycaemic control and weight outcomes in patients with type 2 diabetes. Diabetes Obes Metab.

[22] Inzucchi SE, Tunceli K, Qiu Y, Rajpathak S, Brodovicz KG, Engel SS, Mavros P, Radican L, Brudi P, Li Z, Fan CP, Hanna B, Tang J, Blonde L (2015). Progression to insulin therapy among patients with type 2 diabetes treated with sitagliptin or sulphonylurea plus metformin dual therapy. Diabetes Obes Metab.

[23] Levin P, Wei W, Miao R, Ye F, Xie L, Baser O, Gill J (2015). Therapeutically interchangeable? A study of real-world outcomes associated with switching basal insulin analogues among US patients with type 2 diabetes mellitus using electronic medical records data. Diabetes Obes Metab.

[24] Chitnis AS, Ganz ML, Benjamin N, Langer J, Hammer M (2014). Clinical effectiveness of liraglutide across body mass index in patients with type 2 diabetes in the United States: a retrospective cohort study. Adv Ther.

[25] Davis KL, Tangirala M, Meyers JL, Wei W (2013). Real-world comparative outcomes of US type 2 diabetes patients initiating analog basal insulin therapy. Curr Med Res Opin.

[26] Paul SK, Shaw JE, Montvida O, Klein K (2016). Weight gain in insulin-treated patients by body mass index category at treatment initiation: new evidence from real-world data in patients with type 2 diabetes. Diabetes Obes Metab.

[27] Ma X, Steensma DP, Scott BL, Kiselev P, Sugrue MM, Swern AS (2018). Selection of patients with myelodysplastic syndromes from a large electronic medical records database and a study of the use of disease-modifying therapy in the United States. BMJ Open.

[28] Brixner DI, McAdam-Marx C, Ye X, Lau H, Munger MA (2010). Assessment of time to follow-up visits in newly-treated hypertensive patients using an electronic medical record database. Curr Med Res Opin.

[29] Ashton V, Zhang Q, Zhang NJ, Zhao C, Ramey DR, Neff D, Tershakovec AM, Marrett E (2014). LDL-C levels in US patients at high cardiovascular risk receiving rosuvastatin monotherapy. Clin Ther.

[30] Chopra I, Kamal KM (2014). Factors associated with therapeutic goal attainment in patients with concomitant hypertension and dyslipidemia. Hosp Pract (1995).

[31] Saseen JJ, Ghushchyan V, Kaila S, Allen RR, Nair KV (2013). Maintaining goal blood pressures after switching from olmesartan to other angiotensin receptor blockers. J Clin Hypertens (Greenwich).

[32] Crawford AG, Cote C, Couto J, Daskiran M, Gunnarsson C, Haas K, Haas S, Nigam SC, Schuette R (2010). Prevalence of obesity, type II diabetes mellitus, hyperlipidemia, and hypertension in the United States: findings from the GE centricity electronic medical record database. Popul Health Manag.

[33] Brixner D, Bron M, Bellows B, Ye X, Yu J, Raparla S, Oderda G (2013). Evaluation of cardiovascular risk factors, events, and costs across four BMI categories. Obesity (Silver Spring).

[34] der Sarkissian M, Bhak RH, Huang J, Buchs S, Vekeman F, Smolarz BG, Brett J, Ganguly R, Duh MS (2017). Maintenance of weight loss or stability in subjects with obesity: a retrospective longitudinal analysis of a real-world population. Curr Med Res Opin.

[35] Paul SK, Montvida O, Best JH, Gale S, Pethoe-Schramm A, Sarsour K (2018). Effectiveness of biologic and non-biologic antirheumatic drugs on anaemia markers in 153,788 patients with rheumatoid arthritis: new evidence from real-world data. Semin Arthritis Rheum.

[36] Rajagopalan V, Alemao E, Kawabata H, Solomon D (2014). SAT0069 performance of the Framingham cardiovascular risk prediction model with and without c-reactive protein or erythrocyte sedimentation rate in RA: analysis of us electronic medical records database. Proceedings of the Poster Presentations: Rheumatoid Arthritis - Prognosis, Predictors and Outcome.

[37] Wang J, Mullins CD, Mamdani M, Rublee DA, Shaya FT (2007). New diagnosis of hypertension among celecoxib and nonselective NSAID users: a population-based cohort study. Ann Pharmacother.

[38] Tandon N, Carter C, Haas S, Gunnarsson C (2011). Psy64 Ge Centricity Electronic Medical Records Study: Comorbidities and Biologic Experience Among Patients Receiving Golimumab. Proceedings of the Poster Session I Disease-Specific Study: Systemic Disorders/Conditions.

[39] Patel A, Chan W, Aparasu RR, Ochoa-Perez M, Sherer JT, Medhekar R, Chen H (2017). Effect of psychopharmacotherapy on body mass index among children and adolescents with bipolar disorders. J Child Adolesc Psychopharmacol.

[40] Asche C, Said Q, Joish V, Hall CO, Brixner D (2008). Assessment of COPD-related outcomes via a national electronic medical record database. Int J Chron Obstruct Pulmon Dis.

[41] Patel A, Medhekar R, Ochoa-Perez M, Aparasu RR, Chan W, Sherer JT, Alonzo J, Chen H (2017). Care provision and prescribing practices of physicians treating children and adolescents with ADHD. Psychiatr Serv.

[42] Marelli C, Gunnarsson C, Ross S, Haas S, Stroup DF, Cload P, Clopton P, DeMaria AN (2011). Statins and risk of cancer: a retrospective cohort analysis of 45,857 matched pairs from an electronic medical records database of 11 million adult Americans. J Am Coll Cardiol.

[43] Talal AH, LaFleur J, Hoop R, Pandya P, Martin P, Jacobson I, Han J, Korner EJ (2013). Absolute and relative contraindications to pegylated-interferon or ribavirin in the US general patient population with chronic hepatitis C: results from a US database of over 45 000 HCV-infected, evaluated patients. Aliment Pharmacol Ther.

[44] Montvida O, Shaw J, Atherton JJ, Stringer F, Paul SK (2018). Long-term trends in antidiabetes drug usage in the US: real-world evidence in patients newly diagnosed with type 2 diabetes. Diabetes Care.

[45] Crawford AG, Cote C, Couto J, Daskiran M, Gunnarsson C, Haas K, Haas S, Nigam SC, Schuette R, Yaskin J (2010). Comparison of GE centricity electronic medical record database and national ambulatory medical care survey findings on the prevalence of major conditions in the United States. Popul Health Manag.

[46] (2011). GE Healthcare Systems.

[47] (2016). Centers for Disease Control and Prevention.

[48] (2019). Centers for Disease Control and Prevention.

[49] Centers for Disease Control and Prevention.

[50] Hales C, Carroll M, Fryar C, Ogden C (2017). Prevalence of obesity among adults and youth: United States, 2015-2016. NCHS Data Brief.

[51] Fryar C, Ostchega Y, Hales CM, Zhang G, Kruszon-Moran D (2017). Hypertension prevalence and control among adults: United States, 2015-2016. NCHS Data Brief.

[52] Carroll M, Fryar CD, Nguyen DT (2017). Total and high-density lipoprotein cholesterol in adults: United States, 2015-2016. NCHS Data Brief.

[53] Mendola N, Chen TC, Gu Q, Eberhardt MS, Saydah S (2018). Prevalence of total, diagnosed, and undiagnosed diabetes among adults: United States, 2013-2016. NCHS Data Brief.

[54] Owusu Adjah ES, Montvida O, Agbeve J, Paul SK (2017). Data mining approach to identify disease cohorts from primary care electronic medical records: a case of diabetes mellitus. Open Bioinforma J.

[55] McVeigh KH, Newton-Dame R, Chan PY, Thorpe LE, Schreibstein L, Tatem KS, Chernov C, Lurie-Moroni E, Perlman SE (2016). Can electronic health records be used for population health surveillance? Validating population health metrics against established survey data. EGEMS (Wash DC).

[56] Tatem KS, Romo ML, McVeigh KH, Chan PY, Lurie-Moroni E, Thorpe LE, Perlman SE (2017). Comparing prevalence estimates from population-based surveys to inform surveillance using electronic health records. Prev Chronic Dis.

[57] Casey JA, Schwartz BS, Stewart WF, Adler NE (2016). Using electronic health records for population health research: a review of methods and applications. Annu Rev Public Health.

[58] Koebnick C, Langer-Gould AM, Gould MK, Chao CR, Iyer RL, Smith N, Chen W, Jacobsen SJ (2012). Sociodemographic characteristics of members of a large, integrated health care system: comparison with US census bureau data. Perm J.

